# An *in vitro* batch culture study to assess the fermentation of human milk oligosaccharides by faecal microbiota from healthy and irritable bowel syndrome stool donors

**DOI:** 10.1017/gmb.2025.2

**Published:** 2025-03-20

**Authors:** Patricia Sanz Morales, Anisha Wijeyesekera, M. Denise Robertson, Giles Major, Claire L. Boulangé, Peter Philip James Jackson, Carlos Guillermo Poveda Turrado, Glenn R. Gibson

**Affiliations:** 1 Department of Food and Nutritional Sciences, The University of Reading, Reading, UK; 2 Department of Nutrition, Food and Exercise Sciences, Health & Medical Sciences, University of Surrey, Guildford, UK; 3 Nestlé Institute of Health Sciences, Nestlé Research, Lausanne, Switzerland; 4 Department of Food Science, Purdue University, West Lafayette, IN, USA

**Keywords:** gut microbiome, human milk oligosaccharides, IBS

## Abstract

This study explored the effects of different human milk oligosaccharides (HMOs), solely and in combination, on gut microbiota composition and metabolic activity (organic acid production), using anaerobic *in vitro* batch culture fermenters. The aim was to compare prebiotic effects of HMOs (2’FL, 3’FL, 3’SL, 6’SL, LNT, LNnT, and 1:1 ratio mixes of 2’FL/3’SL and 3’SL/LNT) in faecal samples from irritable bowel syndrome (IBS) donors and healthy controls, and to determine the best-performing HMO in IBS. Fluorescent *in situ* hybridisation coupled with flow cytometry was utilised to study microbiota changes in major colonic genera, and organic acid production was assessed by gas chromatography. IBS donors had different starting microbial profiles compared to healthy controls and lower levels of organic acids. In response to HMOs, there were alterations in both the control and IBS faecal microbiomes. In IBS donor fermenters, *Bifidobacterium*, *Faecalibacterium*, total bacterial numbers, and organic acid production significantly increased post-HMO intervention. When comparing the effect of HMO interventions on the microbiota and organic acid production, a mix of 3’SL/LNT HMOs may be the most promising intervention for IBS patients.

## Introduction

Irritable bowel syndrome (IBS) is a prevalent gastrointestinal (GI) disorder with significant negative impacts on quality of life and high healthcare costs (Mearin et al., 2016). Although there is heterogeneity in studies and diagnostic criteria, global prevalence rates are estimated to be 9.2% (Sperber et al., 2017; 2021). IBS poses a considerable burden on individuals and society. Patients typically present with chronic abdominal pain and altered bowel habits, frequently accompanied by bloating and distension. For many patients, IBS is lifelong, with flares of activity followed by unpredictable periods of remission (Spiegel, 2009). Incidence commonly peaks in the third and fourth decades of life, with females more likely to be affected (Spiller et al., 2007). Symptomatic IBS has been associated with economic impact, including loss of work productivity and absenteeism (Buono et al., 2017; Ma et al., 2021).

To aid IBS diagnosis, the Rome Foundation updated their diagnostic criteria in May 2016, including IBS subtyping (Palsson et al., 2016) with distribution of IBS subtypes found to be 31.5% IBS-D (predominantly with diarrhoea), 29.3% IBS-C (predominantly with constipation), and 26.4% IBS-M (mix of constipation and diarrhoea) (Oka et al., 2020), according to stool consistency using the Bristol Stool Chart (Lacy and Patel, 2017).

In terms of aetiology, IBS is suggested to be a disorder of gut–brain interaction (DGBI) caused by alterations at the mucosal microbiota–host interface leading directly to symptoms (Chadwick et al., 2002; Iribarren et al., 2021). Current treatment options are suboptimal and depend on addressing the patients’ most troublesome symptoms (Khanbhai and Singh Sura, 2013). Since evidence supports a disturbed intestinal microbiota and gut–brain axis (Aziz et al., 2021), microbiota-targeted interventions may benefit some people with IBS, by positively modulating the gut microbiome (Iribarren, 2022). IBS is typically characterised by reduced *Bifidobacterium* spp. (Kerckhoffs et al., 2009) and *Faecalibacterium* spp. (Pittayanon et al., 2019), as well as other butyrate-producing microorganisms (Pozuelo et al., 2015). Studies have previously demonstrated that prebiotic oligosaccharides, such as galactooligosaccharides (GOS), may stimulate gut bifidobacteria and help alleviate symptoms of IBS (Silk et al., 2009).

Breastmilk is known to play a crucial role in the development of infants, providing key nutrients and immunological compounds important for initial protection against pathogens (Bode, Rudloff, et al., 2004; Bode, Kunz et al., 2004; Garwolinska et al., 2018). Among these compounds, human milk oligosaccharides (HMOs) represent the third most important component by mass of breastmilk, after lipids and lactose (Oikonomou et al., 2020). HMOs are complex carbohydrates varying from 3 to 22 monosaccharides with a core structure of lactose (Bode et al., 2004; Bode, 2009). They are candidate prebiotics, considered to be the first prebiotics in life, that specifically stimulate *Bifidobacterium* spp. and have recently gathered interest in the context of adult gut conditions, such as IBS (Palsson et al., 2020; Sanz Morales et al., 2022). Compared to other FODMAPs, HMOs may be better tolerated (Bellini et al., 2020; Iribarren et al., 2021). 2′-Fucosyllactose (2’FL) and lacto-N-neotetraose (LNnT) have been studied in previous trials, with promising results such as support of normal bowel function (Elison et al., 2016; Iribarren et al., 2020; Palsson et al., 2020); however, for context, there are over 150 HMO structures that remain unstudied.

Different HMOs may have distinct effects on the gut microbiome given their structural diversity. Prior reports indicate that individual HMOs vary in their effects on cultured bacterial strains (Marcobal et al., 2010; Yu et al., 2012; Hoeflinger et al., 2015; Ayechu-Muruzabal et al., 2018). HMOs may be metabolised by commensal bacteria, such as *Bifidobacterium, Roseburia*, and *Bacteroides*, and may positively modulate the gut microbiome in both infants and adults (Newburg and Morelli, 2015; Underwood et al., 2015; Pichler et al., 2020).

Few studies have looked at HMOs in the context of IBS in adults, and no study, to date, has compared the diverse gut microbiome responses to HMOs between healthy adults and IBS patient donors. Some HMOs have been safely administered to adults with IBS with minimal side effects compared to traditional prebiotics, such as inulin (Iribarren et al., 2020; Jacobs et al., 2023). A 2’FL/LNnT HMO mix in a 4:1 ratio showed promising results in two previous trials (Iribarren et al., 2020; Palsson et al., 2020), although the mechanism of action that led to improvement of IBS symptoms was not explored. Previous *in vitro* investigations have been conducted on this HMO mix; however, no screening has been published to date to establish performance of other HMOs in comparison (Šuligoj et al., 2020).

This study aimed to assess the changes *in vitro* of the gut microbiome using microbial and metabolic profiling pre- and post-HMO intervention in IBS and healthy controls. To do so, anaerobic batch culture fermentations were conducted with faeces from seven IBS patients and three healthy adult donors, testing six single HMOs and two HMO mixes and including fructooligosaccharide (FOS) as a positive control. FOS is a well-documented prebiotic oligosaccharide which stimulates the growth of *Bifidobacterium* (Olesen and Gudmand-Høyer, 2000; Bouhnik et al., 2004). Gas chromatography (GC) and fluorescent *in situ* hybridisation (FISH) techniques were utilised to analyse modifications in organic acid production and bacterial populations caused by HMOs, solely and in combination, with the objective of determining a candidate prebiotic suitable for addressing IBS.

## Methods

### Human milk oligosaccharides

HMOs 2’FL, 3-fucosyllactose (3’FL), 3-sialyllactose (3’SL), 6-sialyllactose (6’SL), lacto-N-tetraose (LNT), and LNnT were provided by Glycom A/S (Denmark) (batch numbers: CPN6817 1000217 FD, CPN291300215, CPN5115 1000916 FD, and CPN4215 00115, all with > 95% purity). 1.5 g of each HMO was used individually (2’FL, 3’FL, 3’SL, 6’SL, LNT, and LNnT) and in combination (0.75 g plus 0.75 g in a 1:1 ratio of 2’FL/3’SL and 3’SL/LNT), using *in vitro* batch culture vessels (Liu et al., 2016) to give a final concentration of 1% per vessel. This would equate to a 5 g/d daily dose *in vivo*, which is an accepted prebiotic dose (Iribarren et al., 2020).

### Faecal sample preparation

Ethical approval for collecting faecal samples from volunteers was obtained from the University of Reading Research Ethics Committee (UREC 18/02). Fresh faecal samples were provided by three healthy donors free from GI disorders (age 24–27) and seven IBS donors (4 IBS-D, 2 IBS-C, and 1 IBS-M, age 22–64), all females. Donors were not regular users of pre-/probiotic supplements or consumers of live yoghurt and had not consumed antibiotics in the 3 months prior to donating. Samples were collected and placed in an anaerobic jar (O_2_ ≤ 0.1%; CO_2_: 7%–15%) using Thermo Scientific AnaeroGen™ 2.5-L anaerobic sachets (Oxoid, Basingstoke, UK). Samples were used for inoculation within 2 h of production. To form a 10% (w/v) faecal slurry, 20 g of weighed faecal sample was homogenised with 180 mL of anaerobically prepared phosphate-buffered saline at 0.1 mol l*^−1^* (PBS, Oxoid, Hampshire, UK), pH 7.4 for 2 min using a stomacher (Seward, stomacher 80, Worthing, UK) at 240 paddle beats/min. We used faecal inocula to ensure that a full diversity of gut microbes were present in the fermenters.

### Batch culture fermentation

Batch culture fermenters were inoculated with 15 mL of fresh faecal slurry from each donor (Costabile et al., 2014; Guergoletto et al., 2016; Henrique-Bana et al., 2020). First, 135 mL of standard basal nutrient medium (Tzounis et al., 2008) was autoclaved at 121°C for 15 min. Medium was then aseptically added to autoclaved 280-mL vessels. The basal medium (per litre) consisted of: 2 g peptone water, 2 g yeast extract, 0.1 g NaCl, 0.04 g K_2_HPO_4_, 0.04 g KH_2_PO_4_, 0.01 g MgSO_4_.7H_2_O, 0.01 g CaCl.6H_2_O, 2 g NaHCO3, 2 mL Tween 80, 0.05 g hemin, 0.01 mL vitamin K1, 0.5 g L-cysteine-HCl, 0.5 g bile salt, and 4 mL resazurin solution (0.25 g/L). Vessels were then left to gas overnight using oxygen-free N_2_ at a rate of 15 mL/min to achieve anaerobic conditions. Vessels were maintained at 37°C using a circulating water bath. The media were adjusted to pH 6.8 and subsequently maintained between 6.7 and 6.9 using pH controllers (Fermac 260, Electrolab, Tewkesbury, UK) connected to 0.5 M solutions of HCL and NaOH. This pH was selected to mimic conditions of the distal colon (Marcobal and Sonnenburg, 2012; Jackson et al., 2022). Immediately prior to faecal inoculation, 1.5 g of each HMO intervention (2’FL, 3’FL, 3’SL, 6’SL, LNT, LNnT, 2’FL/3’SL mix, and 3’SL/LNT mix) was added to each vessel (1% w/v final concentration). In addition, each fermentation run included a negative control vessel, to which only the faecal slurry was added to the medium, and a positive prebiotic control vessel, to which FOS derived from inulin (Orafti® P95, Beneo, Belgium) (1.5 g) was added as an additional substrate. All vessels were inoculated with 15 mL of faecal slurry (10% w/v) to give a final concentration of 1% faeces (w/v). Baseline samples (T0) were taken immediately post-inoculation, and further samples were collected at 4, 8, 24, and 48 h. Samples T0, T8, T24, and T48 were analysed by GC, and T0 and T24 by flow FISH. Stable pH and anaerobic conditions were maintained throughout.

### Preparation of samples

Samples were aliquoted into 1.5-mL Eppendorfs™ (Fisher Scientific) for gas chromatography (GC) analysis (short-chain fatty acids), and FISH (enumeration of bacteria), 1.5 and 0.75 mL, respectively. For GC, samples were centrifuged at 11,600 g for 10 min before transferring the supernatant into a fresh vial and storing the pellet and supernatant at −20°C. For FISH, samples were centrifuged at 11,600 g for 5 min. After removing the supernatant, the pellet was resuspended in 375 μL of PBS before adding 1125 μL of 4% paraformaldehyde. These samples were then stored at 4°C for 4–8 h before being washed twice with 1 mL of PBS and resuspending the pellet in 150 μL of PBS. Finally, 150 μL of ethanol was added, then the samples were vortexed to homogenise, and stored at −20°C pending analysis.

### Fluorescent in situ hybridisation with flow cytometry (Flow-FISH)

Preparation of samples followed the Grimaldi et al.’s (Grimaldi et al., 2017) protocol. This process was applied to principal groups of gut bacteria (Ford et al., 2018; Pittayanon et al., 2019). As we were particularly interested in quantifying changes in major functionally relevant genera, this was our preferred approach rather than exploring the entire diversity by sequencing. The groups we targeted have been shown to fluctuate in IBS patients compared to controls (Han et al., 2022). Briefly, samples were removed from storage at −20°C and vortexed to redisperse. Then, 75 μL of sample was suspended in 500 μL of PBS before vortexing and centrifuging for 3 min at 15,115 g (consistent for all centrifuging during this process). For permeabilisation of the bacterial cell wall, supernatant was discarded, and the pellets were resuspended in TE-FISH (Tris–HCl 1 M pH 8.0, EDTA 0.5 M pH 8.0, and water at 10:10:80) containing lysozyme (1 mg/mL, 2000 activity Units) and incubated in the dark for 10 min at room temperature. Samples were then re-centrifuged and washed using 500 μL PBS. For *in situ* hybridisation, pellets were resuspended in 150 μL of hybridisation buffer (5 M NaCl, 0.2 M Tris–HCl [pH 8.0], and 0.01% [w/v] sodium dodecyl sulphate, 30% formamide), centrifuged, and resuspended again in 1 mL of hybridisation buffer. Then, 50 μL of this solution was added to each Eppendorf containing 4 μL of the oligonucleotide probe solutions, which were vortexed and incubated overnight at 35°C using heating blocks. Following incubation, 125 μL of hybridisation buffer was added, and Eppendorfs were vortexed and centrifuged. After discarding the supernatant, pellets were resuspended in 175 μL of washing buffer (5 M NaCl, 0.02 M Tris/HCl (pH 8.0), 0.5 M EDTA (pH 8.0), and 0.01% sodium dodecyl sulphate), vortexed to homogenise, and then incubated at 37°C for 20 min in a heating block. The washed pellets were then centrifuged again, resuspended in 300 μL/600 μL of PBS (depending on sample concentration), vortexed, and then stored in the dark at 4°C ready for flow cytometry. Enumeration of bacteria was conducted using an Accuri C6 flow cytometer and analysed using the Accuri C Flow Sampler software (Jackson et al., 2023). Ten oligonucleotide probes were selected for inclusion, targeting a range of functionally relevant bacterial populations (Oliphant and Allen-Vercoe, 2019) (Table 1). Additionally, a mixed 338EUB probe was used to enumerate total bacteria. Bacterial enumerations were then calculated through consideration of flow cytometry reading and PBS dilution. This methodology has been previously used in several studies (Grimaldi et al., 2017; Ryan et al., 2021; Kennedy et al., 2024).

**Table 1.** Oligonucleotide probe sequences and corresponding target species (Grimaldi et al., 2017). These probes are used together in equimolar concentration of 50 ng/μL

Probe name	Sequence (5′ to 3′)	Targeted groups	Reference
Non Eub	ACTCCTACGGGAGGCAGC	Control probe complementary to EUB338	(Wallner et al., 1993)
Eub338‡	GCTGCCTCCCGTAGGAGT	Most bacteria	(Daims et al., 1999)
Eub338II‡	GCAGCCACCCGTAGGTGT	Planctomycetales	(Daims et al., 1999)
Eub338III‡	GCTGCCACCCGTAGGTGT	Verrucomicrobiales	(Daims et al., 1999)
Bif164	CATCCGGCATTACCACCC	*Bifidobacterium* spp.	(Langendijk et al., 1995)
Lab158	GGTATTAGCAYCTGTTTCCA	*Lactobacillus* and *Enterococcus*	(Hermie and Harmsen, 1999)
Bac303	CCAATGTGGGGGACCTT	Most bacteroidaceae and prevotellaceae, some porphyromonadaceae	(Manz et al., 1996)
Erec482	GCTTCTTAGTCARGTACCG	Most of the *Clostridium coccoides*–*Eubacterium rectale* group (Clostridium clusters XIVa and XIVb)	(Franks et al., 1998)
Rrec584	TCAGACTTGCCGYACCGC	*Roseburia* genus	(Walker et al., 2005)
Ato291	GGTCGGTCTCTCAACCC	*Atopobium* cluster	(Harmsen et al., 2000)
Prop853	ATTGCGTTAACTCCGGCAC	Clostridial cluster IX	(Walker et al., 2005)
Fprau655	CGCCTACCTCTGCACTAC	*Faecalibacterium prausnitzii* and relatives	(Hold et al., 2003)
Dsv687	TACGGATTTCACTCCT	*Desulfovibrio* genus	(Devereux et al., 1992)
Chis150	TTATGCGGTATTAATCTYCCTTT	Most of the *Clostridium histolyticum* group (*Clostridium* clusters I and II)	(Franks et al., 1998)

### Gas chromatography

Preparation of samples for GC was carried out in line with the method previously described by Richardson and colleagues (Richardson et al., 1989). Samples were defrosted, centrifuged for 30 seconds at 15,115× *g*, and 1 mL transferred to 100 × 16 mm glass vials, together with 50 μL internal standard (0.1 M 2-ethylbutyric acid), 0.5 mL concentrated HCl, and 3 mL diethyl ether. Vials were vortexed for 1 min and centrifuged for 10 min at 2000× *g* (Eppendorf 5804 R, Stevenage, UK). The upper diethyl ether layer was extracted and transferred to new vials, from which 400 μL was taken and added with 50 μL of N-methyl-N-(tert-butyldimethylsilyl) trifluoroacetamide (MTBSTFA) to screwcap HPLC vials. The vials were protected from light and stored at room temperature for 72 h prior to analysis to allow for all organic acids, including short-chain fatty acids (SCFAs) (acetate, propionate, butyrate, and valerate), branched-chain fatty acids (BCFAs) (isovalerate and isobutyrate), and lactate, to derivatise.

Samples were analysed using a 5690 series Gas Chromatograph (Hewlett Packard, London, UK) with HP-5 ms column (L × I.D. 30 m × 0.25 mm, 0.25 μm film thickness) coating of crosslinked (5%-phenyl)- ethylpolysiloxane (Agilent, Santa Clara, CA, USA). Then, 1 μL of each sample was injected with a run time of 17.7 min. Injector and detector temperatures were 275°C, and the column temperature was programmed from 63°C to 190°C at 5°C per minute and held at 190°C for 3 min. Helium was used as the carrier gas at a flow rate of 1.7 mL/min (head pressure, 133 KPa). A split ratio of 100:1 was used. The external standard solution included acetic acid (30 mM), propionic acid (20 mM), n-butyric acid (20 mM), n-valeric acid (5 mM), iso-butyric acid (5 mM), and iso-valeric acid (5 mM) (all Sigma-Aldrich). Quality control samples of external standard solution were included for every 20 samples to maintain accurate calibration. Peak integration was performed using Agilent Chem Station software (Agilent Technologies, Cheadle, UK), and quantification of each SCFA (mM) was calculated using internal response factors as described previously (Liu et al., 2016).

### Statistical analysis

Statistical Package for Social Science version 27 (SPSS Inc., Chicago, IL, USA) was used for all statistical analyses. To assess changes in bacteriology and organic acid production, a general linear model was constructed to assess repeat measures (Treatment*Time). Comparisons were also performed to assess any potential significant differences between health status within each treatment (Health status*Treatment*Time) at T0 and T24 in both bacteriology and total organic acid production. Post hoc comparisons were also performed to identify any significant differences between organic acid production at 48 h within IBS and healthy treatments. All post hoc pairwise comparisons were corrected for type 1 errors using Bonferroni adjustment within each general linear model. All tests were two-tailed and P values were considered significant at *p* ≤ 0.05 and are displayed by * = *P* ≤ 0.05, ** = *P* ≤ 0.01, and *** = *P* ≤ 0.001. Graphs were generated in GraphPad Prism (version 10.0.2 for Windows, GraphPad Software, Boston, Massachusetts, USA, www.graphpad.com).

## Results

### Enumeration of bacteria with flow FISH

#### Healthy donors

**Figure 1.**
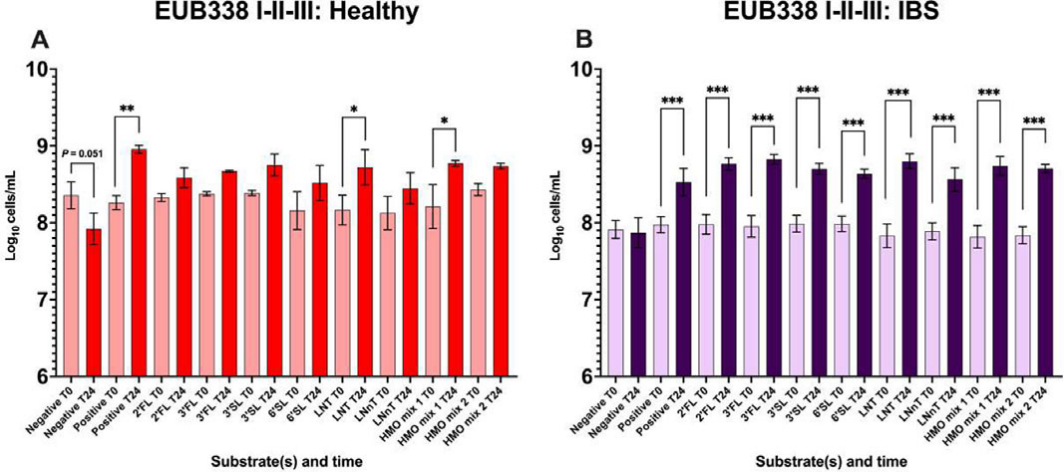
Enumeration of bacteria by flow FISH using samples from anaerobic fermenters with faecal inocula taken at baseline (T0) and following 24 h (T24) of faecal (1%) fermentation within the negative control, positive control, six single HMOs, and two different HMO mixes (2’FL/3’SL and 3’SL/LNT) represented as log_10_ cells/mL culture. (A) Total bacteria for three healthy donors. (B) Total bacteria for seven IBS donors. Significant differences within vessels are displayed as * = *P* ≤ 0.05, ** = *P* ≤ 0.01, and *** = *P* ≤ 0.001. Error bars represent standard deviation (SD).

No significant differences in total bacterial counts (probes Eub338I-III) were observed between vessels at baseline (T0) for three healthy donors. However, there were changes in total bacterial count between baseline and 24 h (T24) (Figure 1A). At peak bacterial growth (T24), growth was significantly higher in the positive control (FOS), LNT, and HMO mix 1 (2’FL/3’SL) interventions (*P* = 0.002, *P* = 0.014, and *P* = 0.013, respectively) than at baseline (Figure 1A). For bacterial subgroups, HMOs impacted growth in diverse ways (Supplementary Figure 1). For instance, bifidobacteria significantly increased after 24 h of fermentation in vessels containing FOS, LNT, and HMO mix 1 (Supplementary Figure 1, A). There were no significant changes between T0 and T24 for other bacterial subgroups, such as Lab158 (*Lactobacillus* and *Enterococcus*) or Prop853 (Clostridial cluster IX) (Supplementary Figure 1, B. G). *Faecalibacterium* significantly decreased with 2’FL and LNnT (Supplementary Figure 1, H).

### IBS donors

No significant differences in bacterial count were observed between vessels at baseline for seven IBS donors. In IBS, all HMO interventions lead to significant increases in total bacterial counts (*P* < 0.001) between T0 and T24 (Figure 1B).

**Figure 2.**
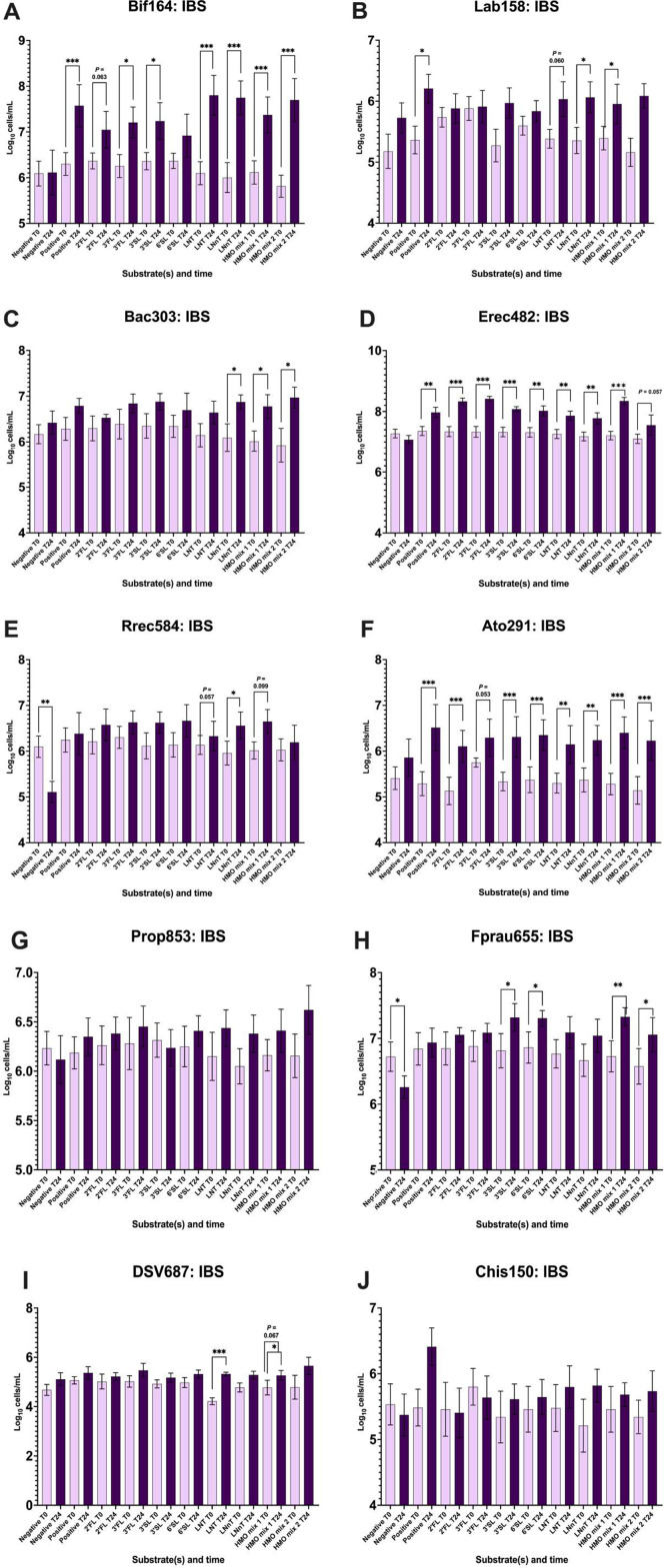
Enumeration of bacterial groups by flow FISH from anaerobic batch fermentation samples with faecal inocula of seven IBS donors at T0 and T24. (A) *Bifidobacterium* spp.; (B) *Lactobacillus* and *Enterococcus*; (C) most Bacteroidaceae and Prevotellaceae, some Porphyromonadaceae; (D) most of the *Clostridium coccoides*–*Eubacterium rectale* group (Clostridium cluster XIVa and XIVb); (E) *Roseburia* genus; (F) *Atopobium* cluster; (G) Clostridial cluster IX; (H) *Faecalibacterium prausnitzii* and relatives; (I) *Desulfovibrio* genus; and (J) most of the *Clostridium histolyticum* group (*Clostridium* cluster I and II). Significant differences are displayed as * = *P* ≤ 0.05, ** = *P* ≤ 0.01, and *** = *P* ≤ 0.001. Error bars represent SD.

Enumeration of bacterial subgroups in IBS showed some increases from T0 to T24 with HMO fermentation. *Bifidobacterium* (Bif164) and *Atopobium* cluster (Ato291) growth were significantly higher in all HMO interventions (*P* < 0.05) (Figure 2A,F,) with the exception of Bif164 with 6’SL (*P* = 0.132) and Ato291 with 3’FL (*P* = 0.053).

*Faecalibacterium* in IBS (Figure 2H) significantly grew in four vessels, containing 3’SL (*P* = 0.021), 6’SL (*P* = 0.041), HMO mix 1 (2’FL/3’SL) (*P* = 0.006), and HMO mix 2 (3’SL/LNT) (*P* = 0.04).

In the *Lactobacillus* and *Enterococcus* bacterial subgroups (Figure 2B), there was significant growth in the positive control (FOS), LNnT, and HMO mix 2. There was significant growth of Bacteroidaceae and Prevotellaceae (Figure 2C) with LNnT and HMO mixes 1 and 2.

Most of the *Clostridium coccoides*–*Eubacterium rectale* group (*Clostridium* cluster XIVa and XIVb) (Figure 2D) responded well to HMOs, causing significant increases except in the HMO mix 2 treatment group. *Roseburia* (Figure 2E) showed a growth trend between T0 and T24 in all HMO interventions, although only LNnT caused a significant increase (*P* = 0.038). Clostridial cluster IX (Figure 2G) increased between T0 and T24, although it did not reach a significant level. *Desulfovibrio* (Figure 2I), a sulphate-reducing genus (Gibson et al., 1988), significantly increased in LNT and HMO mix 1. Finally, the *Clostridium histolyticum* group (*Clostridium* cluster I and II) (Figure 2J) showed no significant changes in response to the positive control (FOS) or HMOs.

### Healthy compared to IBS

**Figure 3.**
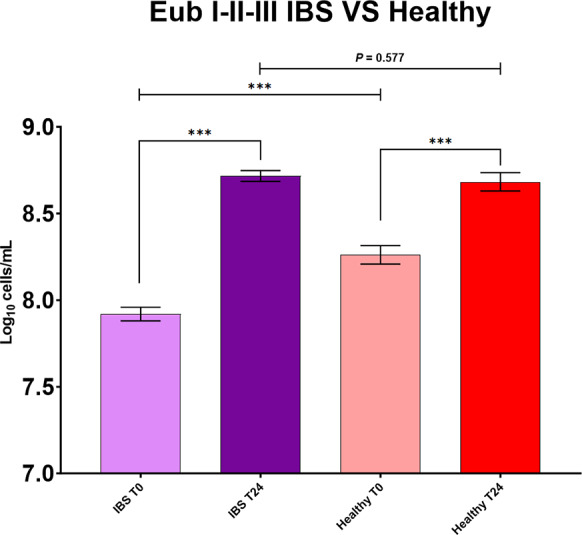
Pairwise comparisons for total bacterial numbers in seven IBS versus three healthy donors for all intervention vessels from anaerobic batch culture fermenters with faecal inocula (positive control and HMOs, excluding negative control vessel). Significant differences are displayed as * = *P* ≤ 0.05, ** = *P* ≤ 0.01, and *** = *P* ≤ 0.001. Error bars represent SD.

**Figure 4.**
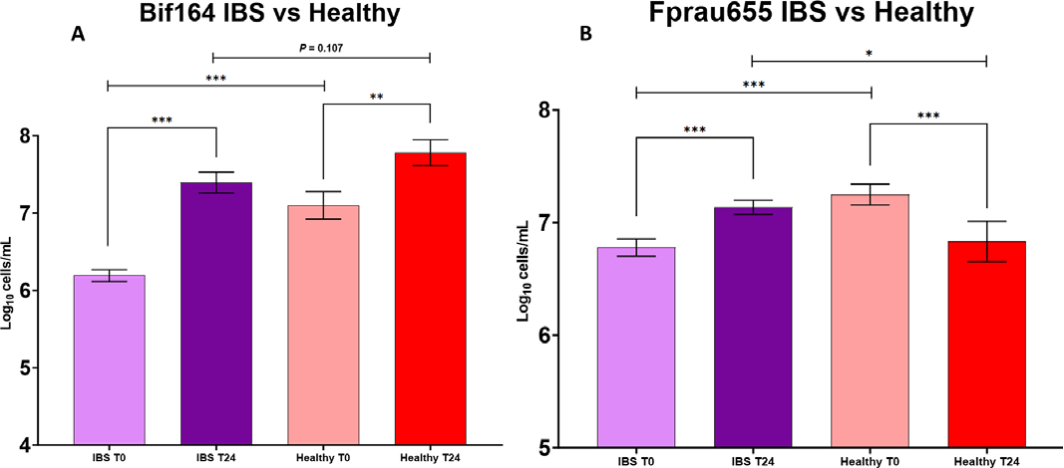
Pairwise comparisons for *Bifidobacterium* spp. (A) and *Faecalibacterium* spp. (B) in samples from anaerobic batch culture fermenters with faecal inocula of seven IBS versus three healthy volunteers at T24. All HMO interventions and positive control (FOS) were included in the analysis, while the negative control was excluded. Significant differences are displayed as * = *P* ≤ 0.05, ** = *P* ≤ 0.01, and *** = *P* ≤ 0.001. Error bars represent SD.

There were significantly lower total bacterial counts in the batch fermenter samples from T0 in IBS compared to healthy (*P* < 0.001); however, levels at T24 were comparable (Figure 3). Moreover, pairwise comparisons highlighted significantly lower baseline *Bifidobacterium* and *Faecalibacterium* levels in IBS compared to healthy donors at T0 (Figure 4). Interestingly, *Faecalibacterium* significantly increased in IBS in response to HMOs, whereas in healthy there was a decrease (Figure 4).

Healthy controls showed lower levels of *C. coccoides*–*E. rectale* group (*Clostridium* cluster XIVa and XIVb) (Erec482) (*P* < 0.001) than in IBS, and higher levels of most of the *C. histolyticum* group (*Clostridium* cluster I and II) (Chis150) (*P* = 0.005) at T0. No significant differences were found between IBS and healthy for other bacterial groups at baseline (Lab158, Bac303, Rrec584, Ato291, Prop853, and Dsv687). At T24, no significant differences were found in *Bifidobacterium* (Bif164), Bacteroidaceae and Prevotellaceae (Bac303), *Atopobium* cluster (Ato291), and Clostridial cluster IX (Prop853) between IBS and healthy. However, there were significantly higher levels of *Lactobacillus* and *Enterococcus* (Lab158, *P* = 0.028), *C. coccoides–E. rectale* group (Erec482, *P* = 0.003), *Roseburia* genus (Rrec584, *P* = 0.013), *Faecalibacterium prausnitzii* (Fprau655, *P* = 0.047), *Desulfovibrio* (Dsv687, *P* < 0.001), and *C. histolyticum* group (Chis150, *P* = 0.011) in IBS compared to healthy at T24.

### Organic acid production

SCFAs, BCFAs, and lactate were measured during fermentation at T0, T8, T24, and T48. Acetate was the most abundant SCFA detected in all fermentations, followed by propionate and butyrate. All SCFAs significantly increased during fermentation, and peak levels of total organic acids were found at T48 (Supplementary Figure 2). Lactate concentrations peaked at T8.

### Healthy donors

**Figure 5.**
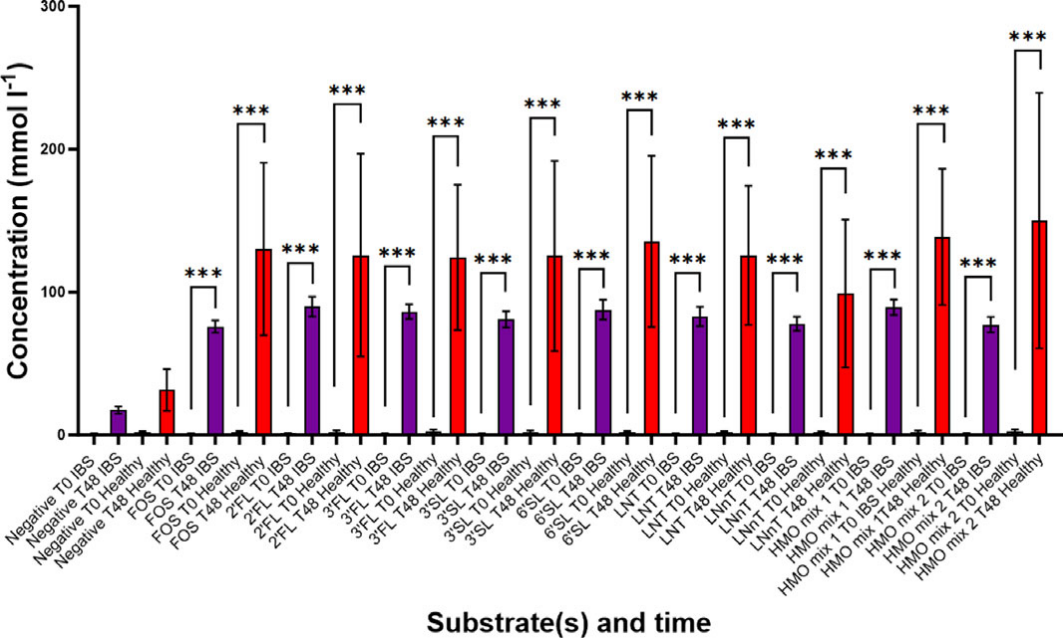
Concentration of total organic acids from anaerobic batch culture fermenters with faecal inocula measured by gas chromatography (in mmol l^−1^) at baseline and 48 h (T0 vs. T48). Total organic acids (expressed as the sum of acetate, propionate, butyrate, isobutyrate, isovalerate, valerate, and lactate) for seven IBS donors (purple bars) and three healthy donors (red bars) are shown in response to the negative control, HMO interventions, and positive control (FOS). Significant differences were tested using paired t-tests and are displayed as *** = *P* ≤ 0.001. Error bars represent SD.

In healthy donors, there were increases in total organic acids (Figure 5), acetate, butyrate, valerate, and propionate (Supplementary Figure 3) production in response to FOS and all HMO interventions, although only significant between T0 and T8 for acetate and propionate in some interventions (acetate: FOS, LNT, and HMO mixes 1 and 2, *P* < 0.05; and propionate: HMO mix 1, *P* < 0.001). The highest concentrations of acetate, butyrate, isobutyrate, isovalerate, valerate, and propionate were reached at T48. Isovalerate significantly increased in the negative control, 3’SL, 6’SL, and HMO mix 1 at T48. Isobutyrate significantly increased in FOS, 3’SL, 6’SL, LNT, and HMO mix 1 at T48. Lactate production tended to increase towards T8 and was not significantly different when comparing to other time points except for FOS at T8 and T48 (*P* = 0.004 and *P* = 0.02, respectively) and HMO mix 1 at T48 (*P* = 0.004) compared to T0.

### IBS donors

**Figure 6.**
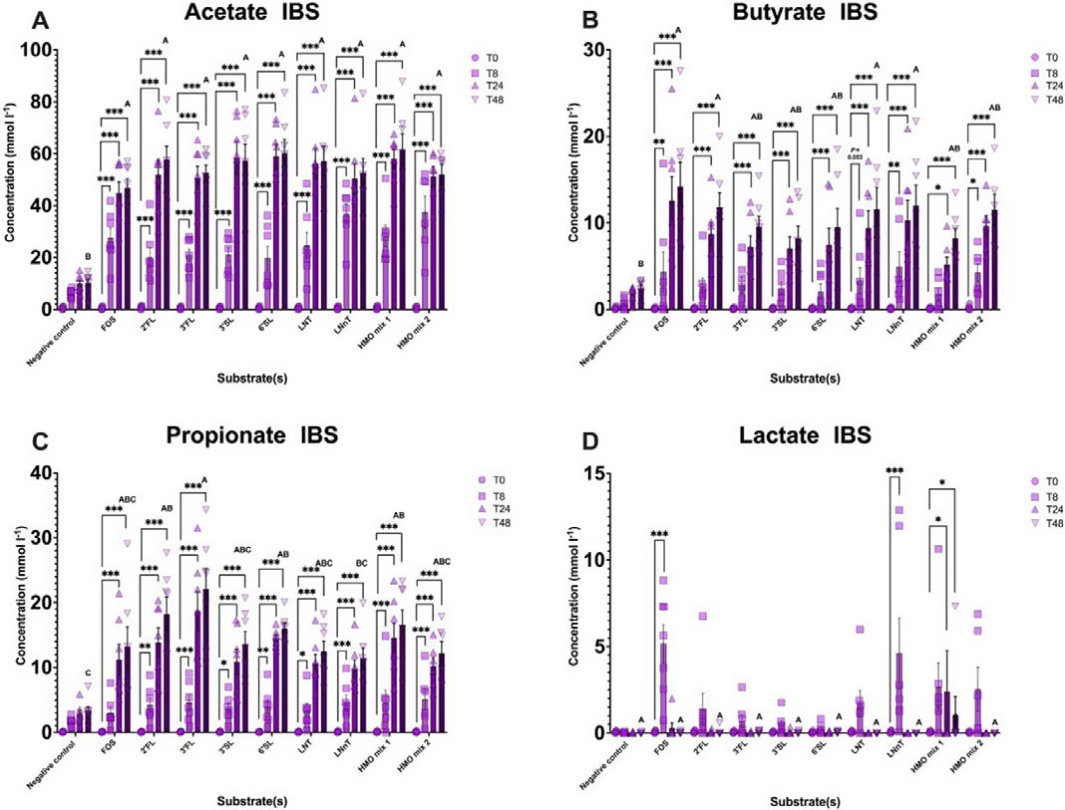
Concentrations of SCFAs and lactate from anaerobic batch culture fermenters with faecal inocula of seven IBS donors measured by gas chromatography (in mmol l^−1^) for seven IBS volunteers in response to different substrates and negative control. Levels of (A) acetate, (B) butyrate, (C) propionate, and (D) lactate at T0, T8, T24, and T48 are colour coded with different shades of purple in the bar plots. Significant differences are displayed as * = *P* ≤ 0.05, ** = *P* ≤ 0.01, and *** = *P* ≤ 0.001. Significant differences (*P* < 0.05) between substrates at T48 are marked with different letters (A, B, and C). Error bars represent SD.

There was a significant increase in total organic acids (Figure 5), acetate, butyrate, and propionate production over 48 h in response to FOS and all HMOs in seven IBS donors. The highest concentration of these SCFAs was reached at T48 (Figure 6A–C). Lactate production peaked at T8 and was only significantly higher than baseline in the vessels containing FOS and LNnT (*P* < 0.001) and at T24 for HMO mix 1 (*P* = 0.022) in IBS (Figure 6D).

Changes in less abundant valerate and BCFAs (isovalerate and isobutyrate) were observed during fermentation of the different substrates (Supplementary Figure 4). Of note, fermentation with HMO mix 2 (3’SL/LNT) tended to yield lower valerate and isovalerate than the other HMO interventions.

### Healthy compared to IBS

**Figure 7.**
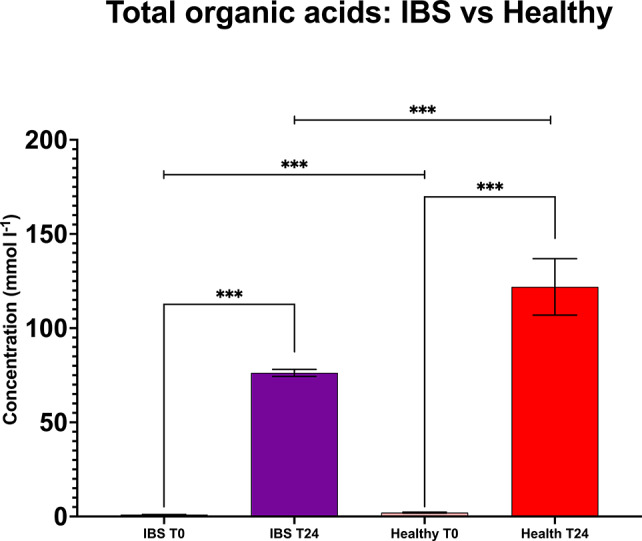
Pairwise comparisons in seven IBS versus three healthy volunteers for total organic acids from anaerobic batch culture fermenters with faecal inocula at baseline and T24. T24 includes all HMOs tested and FOS without the negative control vessel. Significant differences are displayed as * = *P* ≤ 0.05, ** = *P* ≤ 0.01, and *** = *P* ≤ 0.001. Error bars represent SD.

When taking all interventions together, total levels of organic acids were significantly higher in healthy compared to IBS at T0 (*P* < 0.001) and at T24 (*P* < 0.001) (Figure 7). Diving into the separate SCFAs, butyrate, propionate, and acetate were lower in IBS compared to healthy (*P* < 0.001) at T0, as was valerate (*P* = 0.012). The only organic acid that was found to be higher in IBS at T0 was isovalerate (*P* = 0.011). No differences were found between healthy and IBS for lactate and isobutyrate at baseline.

At T24, no significant differences were found between IBS and healthy for butyrate, lactate, isobutyrate, valerate, and isovalerate. However, there were higher levels of propionate and acetate in healthy donors (*P* < 0.001).

## Discussion

This study primarily aimed to evaluate the effect of candidate HMOs or HMO mixes on faecal microbiota for application in patients with IBS. Previous *in vitro* investigations have been conducted on a 2’FL/LNnT HMO mix, however, no screening has been published to date to establish performance of other HMOs in modulating gut microbiota of IBS patients (Šuligoj et al., 2020). Here, we investigated using *in vitro* fermentations a variety of HMOs, which are already being commercially manufactured, and their effects on the gut microbiota in IBS and healthy individuals, to evaluate microbial and metabolic changes.

Batch culture fermenters inoculated with faecal matter from healthy and IBS donors allowed for the study of microbial changes and the production of organic acids in response to different HMOs (Gibson and Fuller, 2000). In addition to a negative control vessel, which allowed for comparison of the HMO vessels to the baseline microbiota, FOS derived from inulin was included as a confirmed prebiotic substrate due to its well-documented effects on *Bifidobacterium* spp. and SCFA production (Meyer and Stasse-Wolthuis, 2009; van der Beek et al., 2018). As expected, fermentation of FOS resulted in a substantial increase in *Bifidobacterium* spp., coupled with significantly increased concentrations of acetate and lactate over the 48-hour period (Figure 4). These results are in line with previous data describing a bifidogenic effect of FOS and therefore provide evidence that the batch culture fermentation models functioned as intended (Meyer and Stasse-Wolthuis, 2009; van der Beek et al., 2018).

A positive effect of HMOs on beneficial bacteria at the expense of other commensal or potential pathogenic bacteria was seen, leading to a shift in the microbiota of IBS and healthy donors. This effect was similar to the positive control (FOS), and in some cases had a greater effect on bacterial growth compared to FOS. With few exceptions (2’FL, 6’SL), beneficial bacteria such as *Bifidobacterium* significantly increased in response to HMOs, whereas opportunistic pathogens such as *Desulfovibrio* and *C. histolyticum* did not (Goldstein et al., 2003). Moreover, increases in *Bifidobacterium* were associated with increases in acetate. *Bifidobacterium* is a well-known acetate producer and may cross-feed other bacteria to produce SCFAs such as butyrate (Fukuda et al., 2011; Hoeflinger et al., 2015; Iribarren, 2022). Although only significant in some treatment groups, there were increases in butyrate-producing microorganisms *Roseburia* (with LNnT) and *Faecalibacterium* (with 3’SL, 6’SL, and HMO mixes 1 and 2) in response to HMOs in IBS. Concomitantly, a rise in butyrate was observed in the same groups. Stool levels of predominant butyrate producers have been shown to correlate with stool SCFA levels (Kumari et al., 2013; Ryan et al., 2021). Further studies should investigate whether stool SCFA could be used as a proxy for butyrate producers in the gut microbiome (De Vuyst and Leroy, 2011; Kumari et al., 2013). Of note, concentrations of lactate peaked at around T8 and fell once again by T24, which was likely a reflection of cross-feeding pathways, where certain bacteria are able to utilise lactate for the production of other SCFAs and metabolites (Louis and Flint, 2017). SCFAs confer multiple biological effects on the gastrointestinal tract. Butyrate is a key energy source for colonocytes, contributing to the integrity of the intestinal barrier and may be immunomodulatory (Lenoir et al., 2020; Siddiqui and Cresci, 2021). Acetate has been linked with multiple benefits, including improved protection against infection (Antunes et al., 2019). These results are relevant for IBS populations since modulating the gut microbial ecosystem by limiting the growth of opportunistic pathogens and enhancing bifidobacteria and SCFA production might improve gut barrier function and mucosal immune system and reduce GI symptoms (Jiang et al., 2022).

When comparing HMO interventions, HMO mix 2 (3’SL/LNT) is of particular interest because it promotes *Bifidobacterium, Lactobacillus*, and *Faecalibacterium* while limiting bacteria that are typically found in dysbiotic microbiomes such as Bacteroidetes and *Clostridium* clusters (Jeffery et al., 2020). In addition, it promoted butyrate production among other SCFAs and limited the production of the BCFA, isovalerate, a marker of proteolytic activity (Diether and Willing, 2019). Increased bacterial proteolysis in adult gut microbiomes has been associated with pro-inflammatory status and may enhance production of potentially uraemic toxins such as p-cresol and indoxyl (Mariaule et al., 2021; Caminero et al., 2023). Thus, HMO mix 2 (3’SL/LNT) is a good candidate to improve gut microbial ecosystems in IBS, which may be beneficial for gut barrier function, reduction in inflammation, and alleviation of IBS-related symptoms. These symptoms are typically gut discomfort, bloating, flatulence, changes in bowel habits, anxiety, depression, sleep disorder, and fatigue (Fletcher et al., 2008). Clinical trials testing HMO mix 2 (3’SL/LNT) are required to better understand the scientific translation of these findings *in vivo*, and evaluate symptom improvement in IBS patients.

When combining all interventions, the comparison between IBS donors and healthy controls showed that SCFA concentrations were lower both at T0 and T48, when the SCFAs peaked. In addition, *Faecalibacterium* and *Bifidobacterium* were lower at T0 in IBS compared to healthy controls (*P* < 0.001) (Supplementary Figures 2 and 3), suggesting that SCFA production could be linked to dysbiosis in IBS (Chassard et al., 2012). Altered levels of SCFA in IBS have previously been reported (Farup et al., 2016; Jiang et al., 2022), and are likely related to a different bacterial ecosystem (Tana et al., 2010; Pozuelo et al., 2015; Gargari et al., 2018). These microbial differences at T0 could also be the reflection of a divergence in dietary intake between IBS and healthy donors. IBS patients often follow a restricted low-fibre diet to alleviate bowel symptoms, which can alter the composition and functions of the microbiome independently of other gastrointestinal disorders (Makki et al., 2018).

Nevertheless, at T24, there were no significant differences between IBS and healthy volunteer bifidobacteria and total bacterial counts (*P* = 0.107, *P* = 0.577), suggesting that experimental conditions allowed for enough time and capacity to increase bacterial numbers and beneficial bacteria to a similar level found in healthy controls, even though these were not entirely reflected in the acetate production.

Limitations of the current experimental setup include diversity in age and IBS subtypes of donors. Moreover, FISH is accurate quantitatively but does not encompass the full diversity that would be evident by next-generation sequencing. In future studies, particularly *in vivo*, both approaches would be an informative combination. Batch culture models provide a controlled, closed system with an equal amount of carbon and nitrogen for bacteria to grow on within each vessel, and the use of a negative control vessel allows for undigested food sources within faeces to be ruled out as responsible for changes over the fermentation period. While we did not determine carbohydrate depletion in the fermenters, we can be confident that microbial fermentation in the test vessels can be attributed to the additional prebiotic or HMO – as evidenced by organic levels in control comparisons. These results remain to be confirmed in a prospective, randomised clinical trial and this is planned.

## Conclusions

This *in vitro* study with different HMOs demonstrated prebiotic effects using batch culture fermentations with healthy adults and IBS donors. All HMO interventions significantly increased SCFA levels and produced distinct shifts in the gut microbiome within 24 h. Specifically in IBS, both *Bifidobacterium* and *Faecalibacterium* levels were restored after fermentation with 3’SL and HMO mixes 1 (2’FL/3’SL) and 2 (3’SL/LNT). These results can inform the evaluation of novel HMO blends as a future therapeutic intervention in IBS but this remains to be confirmed *in vivo.*

## Supporting information

Sanz Morales et al. supplementary material Sanz Morales et al. supplementary material

## Data Availability

Data supporting the results reported in this paper are openly available from The University of Reading Research Data Archive at https://doi.org/10.17864/1947.000507

## Supplementary material

The supplementary material for this article can be found at http://doi.org/10.1017/gmb.2025.2.
