# Supplementary figures and images for: An in vitro batch culture study to assess the fermentation of human milk oligosaccharides by faecal microbiota from healthy and irritable bowel syndrome stool donors
Source: Gut Microbiome (Camb). 2025 Mar 20;6:e4. doi: 10.1017/gmb.2025.2 (PMC12034501; doi:10.1017/gmb.2025.2)

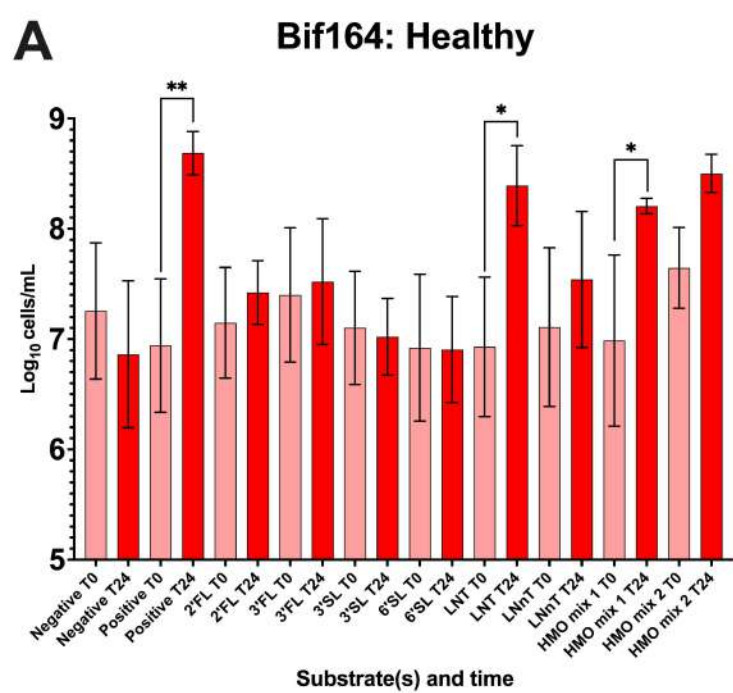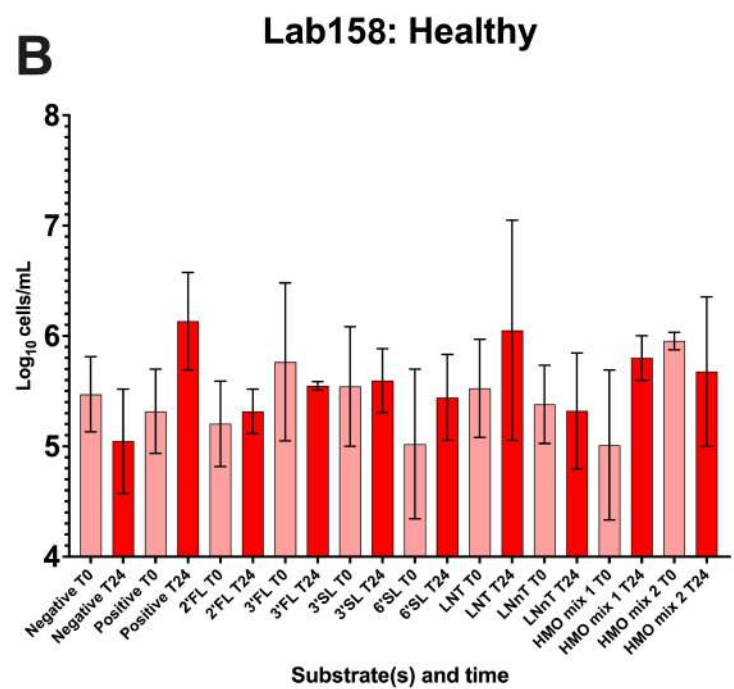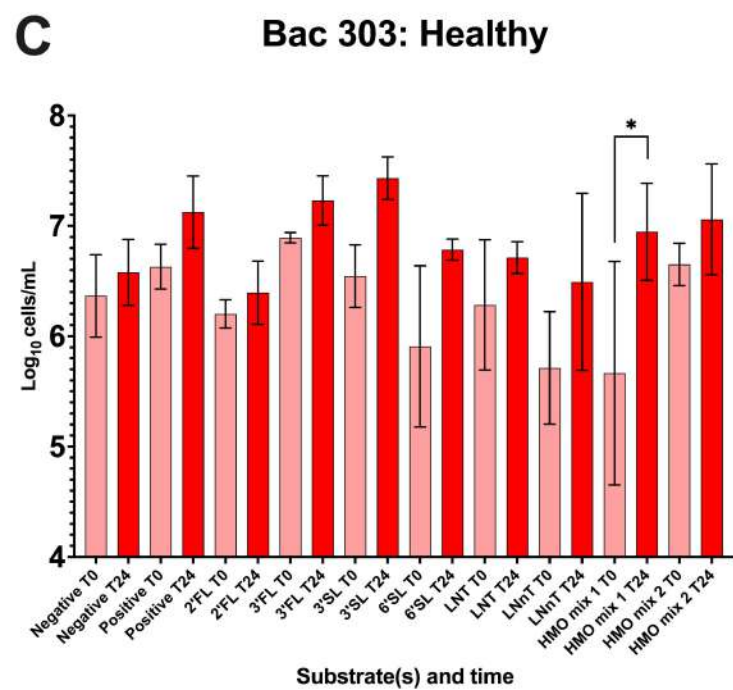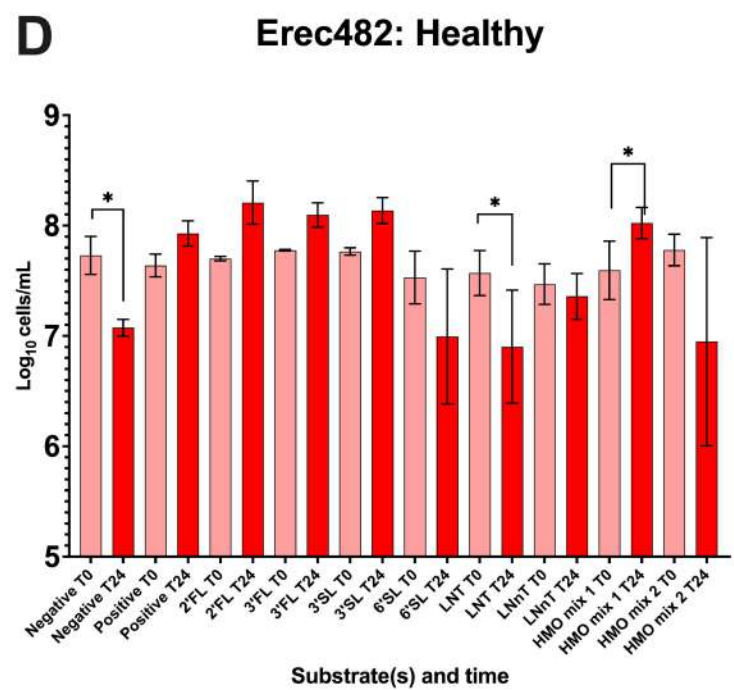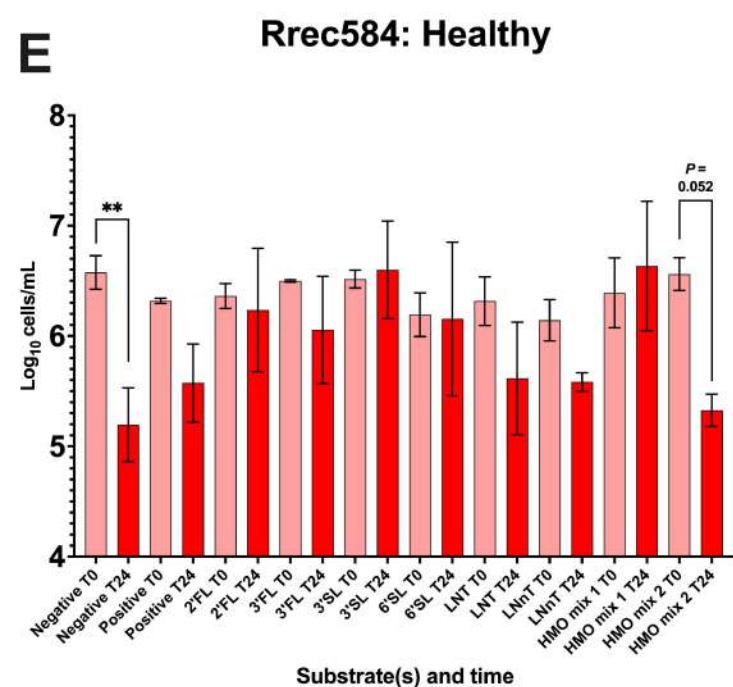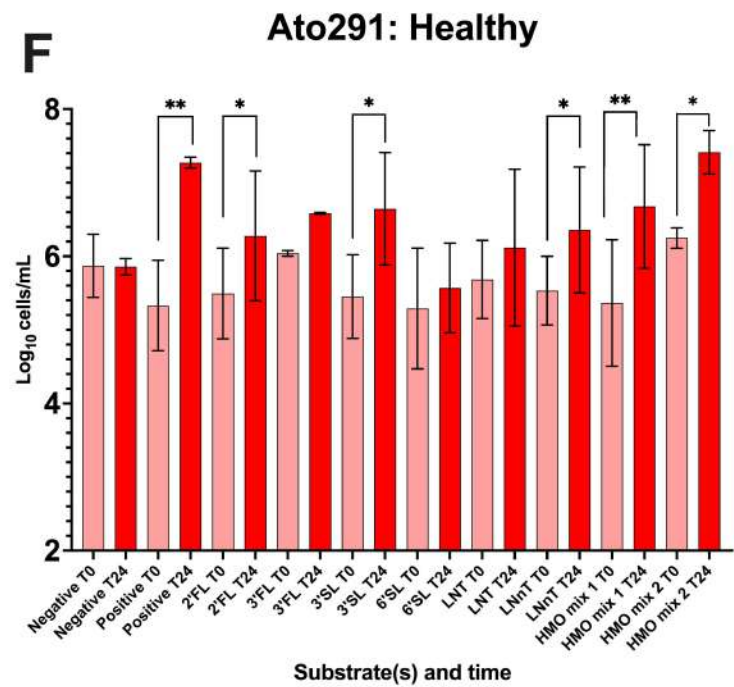

**G** Prop853 Healthy

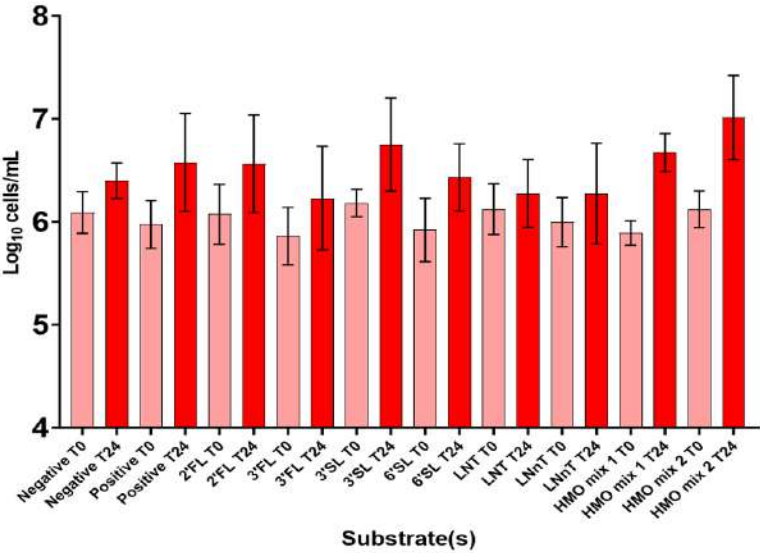

**H** Fprau655 Healthy

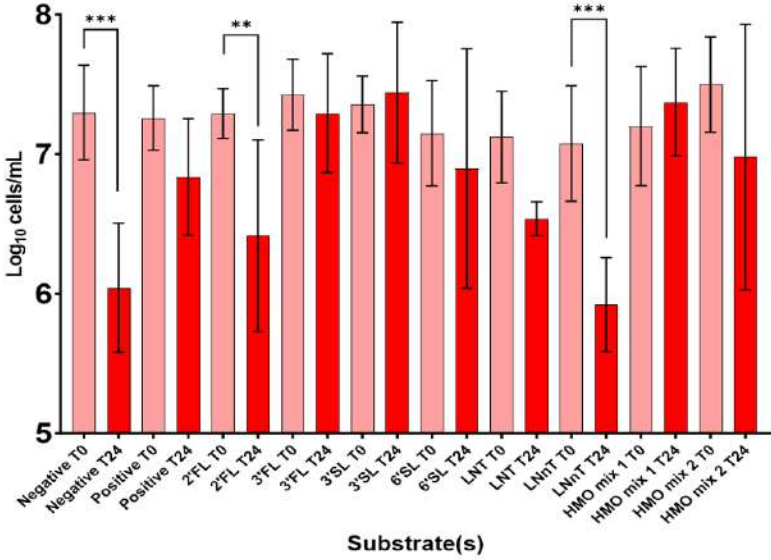

Supplement: Sanz Morales et al. supplementary material [file S2632289725000027sup001.pdf]
